# Nanomaterials in Agriculture: A Pathway to Enhanced Plant Growth and Abiotic Stress Resistance

**DOI:** 10.3390/plants14050716

**Published:** 2025-02-26

**Authors:** Wajid Zaman, Asma Ayaz, SeonJoo Park

**Affiliations:** 1 Department of Life Sciences, Yeungnam University, Gyeongsan 38541, Republic of Korea; wajidzaman@yu.ac.kr; 2 Faculty of Sports Science, Ningbo University, Ningbo 315211, China; asma@nbu.edu.cn

**Keywords:** nanotechnology, plant growth, abiotic stress tolerance, nanomaterials, drought resistance, salinity tolerance, ROS regulation, antioxidant defenses, sustainable agriculture

## Abstract

Nanotechnology has emerged as a transformative field in agriculture, offering innovative solutions to enhance plant growth and resilience against abiotic stresses. This review explores the diverse applications of nanomaterials in agriculture, focusing on their role in promoting plant development and improving tolerance to drought, salinity, heavy metals, and temperature fluctuations. The method classifies nanomaterials commonly employed in plant sciences and examines their unique physicochemical properties that facilitate interactions with plants. Key mechanisms of nanomaterial uptake, transport, and influence on plants at the cellular and molecular levels are outlined, emphasizing their effects on nutrient absorption, photosynthetic efficiency, and overall biomass production. The molecular basis of stress tolerance is examined, highlighting nanomaterial-induced regulation of reactive oxygen species, antioxidant activity, gene expression, and hormonal balance. Furthermore, this review addresses the environmental and health implications of nanomaterials, emphasizing sustainable and eco-friendly approaches to mitigate potential risks. The integration of nanotechnology with precision agriculture and smart technologies promises to revolutionize agricultural practices. This review provides valuable insights into the future directions of nanomaterial R&D, paving the way for a more resilient and sustainable agricultural system.

## 1. Introduction

Nanotechnology has emerged as a revolutionary discipline that provides unprecedented solutions to address pressing challenges across various fields, including agriculture [1]. In recent years, integrating nanotechnology into agriculture has opened new frontiers, offering the potential to enhance plant growth, improve crop resilience to environmental stresses, and ensure sustainable agricultural practices [2]. Due to their nanoscale size, high surface-area-to-volume ratio, and unique physicochemical properties, nanomaterials possess remarkable capabilities that distinguish them from conventional materials [3]. These properties include enhanced reactivity, controlled release, and targeted delivery, making nanomaterials ideal for applications such as nutrient delivery, stress mitigation, and plant pathogen control [4]. As agriculture faces mounting challenges from a growing global population, climate change, and resource limitations, nanotechnology presents a timely and transformative approach to achieving food security and environmental sustainability [5].

The unique properties of nanomaterials enable them to interact with plants at the cellular and molecular levels, significantly influencing physiological and biochemical processes [6]. Metallic NPs, carbon-based nanomaterials, and polymeric composites are among the diverse nanomaterials employed in agricultural practices. Their small size facilitates easy uptake through plant roots or foliage, allowing for efficient transport to various tissues where they enhance nutrient assimilation and metabolism [7]. This capability holds immense potential for addressing agricultural challenges, including nutrient deficiencies, poor soil quality, and susceptibility to abiotic stresses, such as drought and salinity [8]. Additionally, nanomaterials aid in the detoxification of heavy metals in contaminated soils, enabling plants to thrive under suboptimal conditions [9]. The ability of nanomaterials to improve photosynthetic efficiency, enhance antioxidant responses, and regulate hormonal pathways further highlights their potential to revolutionize plant growth and productivity [10].

Agricultural systems today face significant challenges such as climate-induced stresses, declining soil fertility, and water scarcity, all of which threaten global food production. Conventional agricultural practices often depend on chemical fertilizers and pesticides, which pose substantial environmental and health risks [11]. Nanotechnology offers a sustainable alternative by enabling precise, efficient, and eco-friendly solutions. For example, nanomaterial-based fertilizers with controlled nutrient release minimize nutrient runoff while enhancing nutrient uptake efficiency [12]. Similarly, nanosensors integrated into farming systems enable real-time monitoring of soil and plant health, facilitating precision agriculture practices. These advances not only reduce resource wastage but also lower the ecological footprint of agricultural activities [13]. However, the long-term ecological and health impacts of nanomaterials must be rigorously studied to ensure their safe application [14].

The relevance of nanotechnology in agriculture lies in its capacity to bridge the gap between increasing food demands and sustainable production practices. By leveraging nanomaterials to mitigate abiotic stresses such as drought, salinity, and temperature fluctuations, researchers have achieved significant improvements in plant stress tolerance [15]. For instance, nanomaterials enhance the regulation of reactive oxygen species (ROS) and bolster antioxidant defenses, protecting plants from oxidative damage under stress conditions [10]. Additionally, nanomaterials play a crucial role in detoxifying heavy metals and supporting hormonal balance, further underscoring their multifaceted applications in stress adaptation [16]. As research advances, integrating nanotechnology with emerging technologies, such as the Internet of Things (IoT) and artificial intelligence (AI), promises to redefine modern agricultural practices. These synergies are poised to enable precision farming, enhance crop resilience, and contribute to a more sustainable and productive agricultural future [17].

While previous reviews on the application of nanotechnology in agriculture have focused extensively on the role of nanomaterials in enhancing plant growth and tolerance to abiotic stress, this review differentiates itself by providing a comprehensive and balanced perspective on both the benefits and potential environmental risks associated with nanomaterial use. For example, El-Saadony et al. (2022) examined how nanomaterials enhance plant stress tolerance by modulating reactive oxygen species (ROS) regulation and strengthening antioxidant defenses in crops under abiotic stress conditions [2]. Additionally, Wahab et al. (2024) highlighted eco-friendly nanomaterial synthesis methods and their role in sustainable agriculture practices, emphasizing the importance of soil health and food safety in agricultural systems [5]. Unlike many existing reviews, we explore the ecological implications of nanomaterial application, including environmental persistence, toxicity to non-target organisms, and the need for sustainable nanomaterial development. Additionally, we highlight the integration of nanotechnology with precision agriculture, emphasizing its potential for improving resource use efficiency while minimizing environmental impacts. This review also focuses on the development of eco-friendly nanomaterials, offering novel insights into mitigating the risks associated with their application. By addressing these areas, our review provides a more holistic and integrated approach, bridging the gap between nanomaterial application, agricultural productivity, and environmental sustainability. The purpose of this review is to provide a comprehensive overview of the applications of nanomaterials in agriculture, with a particular emphasis on their role in enhancing plant growth and resistance to abiotic stress, as well as the environmental sustainability of their use.

## 2. Types and Characteristics of Nanomaterials in Agriculture

Nanomaterials have diverse structures and compositions, making them highly versatile for agricultural applications. Their classification is based on composition, shape, size, and functionality, which determine their interactions with plant systems [18]. Broadly, nanomaterials used in agriculture can be categorized into metallic NPs, carbon-based nanomaterials, and polymeric nanomaterials, each exhibiting unique properties that make them suitable for specific applications. These materials, through their enhanced physical, chemical, and biological interactions with plants, provide solutions for improved nutrient delivery, stress tolerance, and pest management [19,20]. Understanding the classification and properties of nanomaterials is critical for optimizing their application in plant sciences and addressing specific agricultural challenges [21].

### 2.1. Classification of Nanomaterials

Nanomaterials can be classified based on their composition and functionality. The three primary categories of nanomaterials in agriculture include metallic NPs, carbon-based nanomaterials, and polymeric nanomaterials. Each of these types of nanomaterials exhibits distinct characteristics that influence their effectiveness in different agricultural applications.

#### 2.1.1. Metallic NPs

Metallic NPs, such as silver (Ag), gold (Au), zinc oxide (ZnO), and iron oxide (Fe_2_O_3_), are among the most extensively studied nanomaterials because of their multifaceted agricultural applications. These NPs exhibit exceptional antimicrobial, catalytic, and nutrient-delivery properties [22]. For example, Ag NPs are highly effective in controlling plant pathogens, whereas ZnO NPs enhance the availability of micronutrients, thereby stimulating plant growth and development [23]. Similarly, Fe_2_O_3_ NPs improve plant iron uptake and chlorophyll synthesis—both critical for photosynthesis. The high reactivity and controlled release behavior of metallic NPs make them indispensable for targeted agricultural interventions [24].

#### 2.1.2. Carbon-Based Nanomaterials

Carbon-based nanomaterials, such as carbon nanotubes (CNTs), graphene oxide, and fullerenes, are gaining prominence in agricultural applications because of their structural stability and ability to influence plant physiological processes [25]. CNTs, for instance, enhance water and nutrient uptake by creating nanochannels in root cell membranes, thereby improving plant vigor under drought conditions [26]. Graphene oxide has demonstrated the ability to modulate oxidative stress in plants and facilitate the efficient delivery of agrochemicals. Additionally, the unique electrical and mechanical properties of carbon-based nanomaterials make them highly suitable for biosensing applications, enabling real-time monitoring of plant health and environmental conditions [27].

#### 2.1.3. Polymeric Nanomaterials and Composites

Polymeric nanomaterials, synthesized from biocompatible and biodegradable polymers, are increasingly being utilized in sustainable agriculture because of their ability to encapsulate nutrients, pesticides, or growth regulators, ensuring slow and targeted release to plants [19]. Polymeric composites, which combine organic and inorganic components, further enhance functionality by integrating the strengths of both materials [28]. For instance, chitosan-based NPs are biodegradable and possess antimicrobial properties, making them ideal for environmentally friendly pest management. These polymeric systems not only reduce the ecological footprint of agricultural practices but also maintain or enhance crop productivity [29]. Table 1 summarizes the classification and key properties of nanomaterials used in plant sciences, as compiled from multiple studies.

#### 2.1.4. Comparison of Nanomaterial Types

Each type of nanomaterial—metallic NPs, carbon-based nanomaterials, and polymeric nanomaterials—offers distinct advantages for agricultural applications, and understanding their differences is essential for optimizing their use in plant sciences [35]. Metallic nanoparticles, such as zinc oxide (ZnO) and silver (Ag) NPs, are widely recognized for their high reactivity and efficient nutrient delivery capabilities. They excel in controlling plant pathogens and enhancing micronutrient availability [36]. However, these materials are often associated with environmental persistence and potential toxicity to non-target organisms, raising concerns about their long-term use in agricultural systems [37]. On the other hand, carbon-based nanomaterials, such as carbon nanotubes (CNTs) and graphene oxide, provide mechanical stability and electrical conductivity, which make them particularly useful for improving plant structural integrity and enhancing water and nutrient uptake [38]. These nanomaterials also offer significant advantages in biosensing and monitoring applications. They are more beneficial in drought resistance and oxidative stress regulation but may not provide the same level of micronutrient delivery as metallic NPs [39]. Polymeric nanomaterials offer a more sustainable and eco-friendly approach, with advantages such as biodegradability and slow-release capabilities. While they do not provide the same antimicrobial or pathogen-fighting properties as metallic NPs, they are ideal for targeted nutrient delivery and pest management in environmentally conscious agricultural practices [40]. Additionally, polymeric composites, which combine organic and inorganic components, enhance the overall functionality and adaptability of these materials in different agricultural contexts. The primary advantage of polymeric nanomaterials is their minimal environmental impact and biodegradability, but their effectiveness in stress tolerance and nutrient delivery is typically more gradual compared to metallic and carbon-based nanomaterials [41].

### 2.2. Key Properties of Nanomaterials Relevant to Plant Sciences

#### 2.2.1. Surface Area and Reactivity

The nanoscale dimensions of these materials result in an exceptionally high surface-area-to-volume ratio, which significantly enhances their reactivity and interactions with plant systems. This property facilitates efficient nutrient and agrochemical adsorption, improving their transport and bioavailability to plants [42]. For example, the high reactivity of ZnO NPs ensures the rapid release of Zn ions, which are critical for enzyme activation and chlorophyll production [43]. Moreover, the catalytic properties of metallic NPs, such as Ag and Au, enable them to regulate biochemical reactions in plants, thereby promoting growth and enhancing stress resilience [44].

#### 2.2.2. Size and Solubility

The nanoscale dimensions of nanomaterials enable their penetration through plant cell walls, facilitating the efficient delivery of nutrients and agrochemicals to target sites [45]. NPs in the size range of 1–100 nm are particularly effective at overcoming physical barriers such as the cuticle and root epidermis [46]. Additionally, the solubility of nanomaterials in aqueous and soil environments is critical for determining their bioavailability [47]. For example, highly soluble materials like Fe_2_O_3_ NPs readily release bioavailable iron to plants, effectively addressing iron deficiencies in alkaline soils [48]. Conversely, engineered nanomaterials with controlled solubility can provide a sustained release of nutrients or pesticides, thus reducing the frequency of application and minimizing environmental impacts [49].

By leveraging their unique classifications and properties, nanomaterials offer transformative solutions for agriculture, enabling precise and efficient strategies to enhance crop productivity and resilience [50]. A comprehensive understanding of their types and characteristics is essential for optimizing their integration into sustainable farming systems.

## 3. Mechanisms of Nanomaterial–Plant Interactions

Nanomaterials interact intricately with plants, profoundly influencing their physiology, metabolism, and overall health. These interactions begin with uptake through plant roots or leaves and extend to transport across various tissues [51]. Once internalized, nanomaterials can modify cellular structures and molecular processes to enhance nutrient absorption, stress tolerance, and growth [6]. A thorough understanding of these mechanisms is crucial for optimizing nanotechnology applications in agriculture while mitigating environmental risks and phytotoxicity [52].

### 3.1. Nanomaterial Uptake and Transport

**Figure 1:**
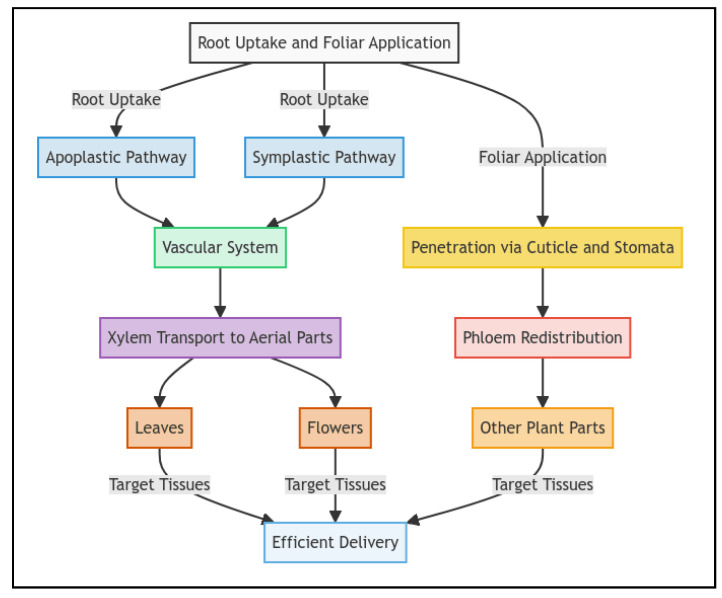
Pathways of nanomaterial interactions with plant roots and tissues.

Nanomaterials primarily enter plants via root uptake or foliar application, after which they are transported to target tissues. Root uptake occurs via the apoplastic and symplastic pathways [53]. In the apoplastic pathway, nanomaterials move through cell wall spaces, whereas in the symplastic pathway, they enter the cytoplasm and travel via plasmodesmata. Once within root tissues, nanomaterials reach the vascular system and are transported upward through the xylem to aerial parts of the plant, such as leaves and flowers [54]. Once inside the plant, nanomaterials are transported through vascular tissues, primarily the xylem and phloem, depending on the mode of application and the physicochemical properties of the nanomaterials. In the xylem, nanomaterials travel via transpiration, benefiting from the plant’s water uptake. Foliar application, on the other hand, involves nanomaterials penetrating the cuticle and stomata of the leaf, bypassing the soil-related barriers and entering the phloem for distribution to non-photosynthetic tissues like roots or fruits [55]. The detailed pathways—from root uptake to transport through the xylem and phloem—are visualized in Figure 1.

#### 3.1.1. Root Uptake and Foliar Application

Plant roots are the primary entry point for nanomaterials in soil-based systems. NPs penetrate root tissues via the apoplastic or symplastic pathways, and the route is determined by size, charge, and surface properties [56]. The apoplastic pathway involves passive transport through cell wall spaces, whereas the symplastic pathway requires active entry through plasmodesmata and depends on metabolic energy [57]. Once within the root cortex, nanomaterials are transported to the xylem for upward movement to aerial parts of the plant [58]. Studies have indicated that metallic NPs such as Ag and ZnO efficiently penetrate root cells, enhancing nutrient uptake and promoting root growth under nutrient-deficient conditions [59].

Foliar application is another effective route for delivery of nanomaterials, particularly to pesticides and growth regulators [60]. Nanomaterials applied to leaves penetrate the cuticle and stomata, bypassing soil-related barriers [61]. For example, carbon-based nanomaterials such as graphene oxide exhibit strong adhesion and penetration properties, enabling the direct delivery of nutrients or agrochemicals to photosynthetically active tissues [62]. This method provides a faster mode of action, reducing the losses caused by leaching or degradation in soil [61].

#### 3.1.2. Translocation Within Plant Tissues

After uptake, nanomaterials are translocated within plants through vascular tissues, primarily the xylem and phloem, depending on their functional properties and the site of application [63]. NPs absorbed through roots typically move via the xylem, driven by transpiration flow [64]. In contrast, foliar-applied NPs are transported through the phloem to non-photosynthetic tissues such as roots and developing fruits [63]. The movement and distribution of nanomaterials depend on their size, solubility, and interactions with plant biopolymers like lignin and cellulose [65]. Smaller NPs are more likely to reach distal tissues, whereas larger aggregates tend to remain localized in specific regions. This targeted distribution enables the precise delivery of nutrients or biocides to areas where they are most needed [66].

### 3.2. Effects at Cellular and Molecular Levels

#### 3.2.1. Influence on Cell Structure

Nanomaterials can influence the structural integrity of plant cells in both beneficial and potentially harmful ways. For instance, metallic NPs like silver (Ag) NPs improve the rigidity and resilience of cell walls, enhancing resistance to microbial invasion and mechanical stress [67]. Ag NPs deposit within the cell wall, increasing its rigidity and resistance to microbial invasion. However, excessive accumulation of metallic NPs within plant cells may disrupt the cell wall structure and permeability, leading to oxidative stress and potential damage to cellular components like the plasma membrane and mitochondria [68,69]. In contrast, carbon-based nanomaterials such as CNTs help plants by modulating water transport and improving nutrient uptake, especially under stress conditions like drought [70].

**Figure 2:**
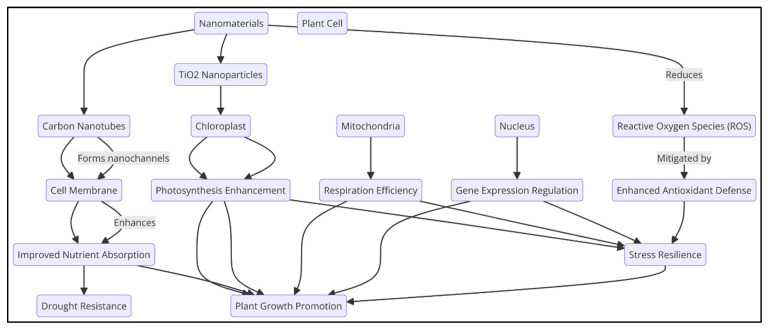
Mechanisms of interactions of nanomaterials with plant cells and their role in enhancing stress resilience.

At the intracellular level, nanomaterials interact with organelles such as chloroplasts, mitochondria, and nuclei, affecting essential processes like photosynthesis, respiration, and gene expression [71]. For example, TiO_2_ NPs enhance chlorophyll production and light absorption, boosting photosynthetic efficiency [72]. Carbon-based nanomaterials like CNTs help to improve cell membrane permeability and enhance nutrient absorption by forming nanochannels within the cell membrane, thus improving cellular functions under drought conditions [70]. By enhancing antioxidant defenses and reducing ROS accumulation, nanomaterials mitigate oxidative stress within plant cells. This contributes significantly to the plant’s stress resilience, especially under adverse environmental conditions [73]. Figure 2 illustrates how nanomaterials such as carbon nanotubes (CNTs) and TiO_2_ nanoparticles interact with various plant cell structures, such as the cell membrane, chloroplasts, and mitochondria, influencing processes like photosynthesis, respiration, and stress resilience.

#### 3.2.2. Impact on Nutrient Absorption and Metabolism

Nanomaterials significantly improve nutrient absorption and metabolism in plants, enhancing growth and stress tolerance [74]. Metallic NPs, such as ZnO and Fe_2_O_3_, release essential micronutrients that plants readily absorb and utilize. These NPs bypass traditional diffusion barriers and directly deliver nutrients to metabolic activity sites [75]. Enhanced nutrient uptake supports enzymatic functions, chlorophyll synthesis, and energy production, ultimately increasing plant vigor and productivity [76].

At the metabolic level, nanomaterials modulate critical pathways related to energy production and stress responses [77]. For instance, NPs enhance nitrogen metabolism by increasing nitrate reductase activity, leading to improved protein synthesis and biomass accumulation [78]. Additionally, carbon-based nanomaterials like graphene oxide reduce ROS accumulation, minimize oxidative stress, and promote metabolic stability under adverse conditions [79].

## 4. Impact of Nanomaterials on Plant Growth

Nanomaterials have a significant impact on various stages of plant growth, from seed germination to mature development [80]. Their unique physicochemical properties enable interactions with plants at critical growth stages, enhancing physiological processes, photosynthetic efficiency, and nutrient metabolism. These effects often result in improved biomass production and overall plant health, making nanotechnology a promising tool for addressing agricultural challenges [7,77]. For example, nanoparticles like nitric-oxide-releasing polymeric nanoparticles have been shown to improve soybean seed germination and nodule development, contributing to better crop performance [81]

### 4.1. Enhancement of Germination and Seedling Growth

#### 4.1.1. Benefits in Seed Germination

Nanomaterials play a crucial role in enhancing seed germination by improving water absorption and activating metabolic pathways essential for seedling emergence [82]. Metallic NPs such as ZnO and Fe_2_O_3_ increase germination rates in various crops by promoting enzymatic activity and energy production within seeds [83]. The nanoscale size of these particles enables penetration into seed coats, facilitating nutrient mobilization and enhancing cellular respiration [84]. For example, TiO_2_ NPs improve water uptake and the breakdown of stored carbohydrates in seeds, resulting in faster and more uniform germination [85].

#### 4.1.2. Impact on Root and Shoot Development

In the early stages of growth, nanomaterials profoundly influence root and shoot development, laying a strong foundation for plant growth [86]. ZnO NPs, for example, stimulate root elongation and lateral root formation by increasing the availability of essential micronutrients [87]. Similarly, Ag NPs promote shoot growth by enhancing cell division and elongation in the apical meristem [88]. Carbon-based nanomaterials, such as CNTs, form nanochannels in root cell membranes, facilitating the transport of water and nutrients, thereby supporting robust root and shoot development during seedling growth [89].

### 4.2. Photosynthetic Efficiency and Biomass Production

#### 4.2.1. Improved Photosynthetic Efficiency

Nanomaterials significantly enhance photosynthesis, which is the primary driver of plant growth [90]. TiO_2_ NPs increase light absorption and chlorophyll content in plants, resulting in higher photosynthetic rates. These NPs interact with chloroplasts to improve the efficiency of light-dependent reactions and carbon fixation [91]. Additionally, carbon-based nanomaterials like graphene oxide act as carriers for nutrients such as magnesium, a critical component of chlorophyll, further boosting photosynthetic activity [45]. Enhanced photosynthesis improves energy production, which is essential for sustaining growth and development under optimal and stress conditions [92].

#### 4.2.2. Increase in Biomass Production

The application of nanomaterials often leads to significant increases in plant biomass due to enhanced photosynthesis and nutrient availability [7]. For instance, Fe_2_O_3_ NPs were reported to improve root and shoot biomass in crops such as wheat and rice by facilitating efficient iron uptake and utilization [93]. Similarly, polymeric nanomaterials designed for slow-release fertilizers provide a sustained nutrient supply, reducing growth limitations and promoting biomass accumulation [94]. The ability of nanomaterials to boost biomass production is particularly beneficial for achieving higher crop yields under resource-limited conditions [95].

### 4.3. Impact on Nutrient Uptake and Metabolism

#### 4.3.1. Influence on Nutrient Absorption

Nanomaterials enhance nutrient uptake by increasing the bioavailability of essential elements in soil and facilitating their transport within plant tissues [96]. For instance, ZnO NPs improve Zn ion availability, which is essential for enzyme function and protein synthesis [97]. Similarly, Fe_2_O_3_ NPs address iron deficiency, particularly in calcareous soils, by providing a readily absorbable source of iron. By bypassing traditional barriers to nutrient absorption, such as low soil mobility and insolubility, these NPs ensure that plants receive adequate nutrition for optimal growth and development [98]. Nanomaterials interact synergistically with traditional agricultural practices such as fertilization and irrigation to enhance nutrient uptake. For example, when applied in conjunction with fertilizer, nanomaterials like ZnO NPs can enhance the availability of nutrients like zinc, improving plant nutrition and promoting growth under nutrient-limited conditions [99]. Additionally, nanomaterials can be integrated with irrigation systems to improve water retention and nutrient solubility, ensuring more efficient use of fertilizers and water, thereby reducing overall input costs. These synergies help optimize nutrient management and resource utilization, promoting sustainable farming practices [100].

#### 4.3.2. Effects on Plant Metabolism

Nanomaterials significantly influence plant metabolism by regulating key enzymatic activities and biochemical pathways [101]. For example, the application of nanomaterials enhances nitrate reductase activity and improves nitrogen assimilation and protein synthesis [102]. Metallic NPs, such as Cu and Zn NPs, boost enzymatic activities related to carbohydrate metabolism, ensuring a sufficient energy supply for growth [103]. Carbon-based nanomaterials such as CNTs reduce oxidative stress by scavenging ROS, thereby stabilizing metabolic processes under challenging environmental conditions [104].

Thus, nanomaterials play a transformative role in enhancing plant growth, from germination to biomass accumulation, by optimizing physiological, biochemical, and metabolic functions (Table 2). These advantages underscore their potential as a key technology for advancing sustainable agricultural practices [105].

## 5. Role of Nanomaterials in Enhancing Abiotic Stress Tolerance

**Figure 3:**
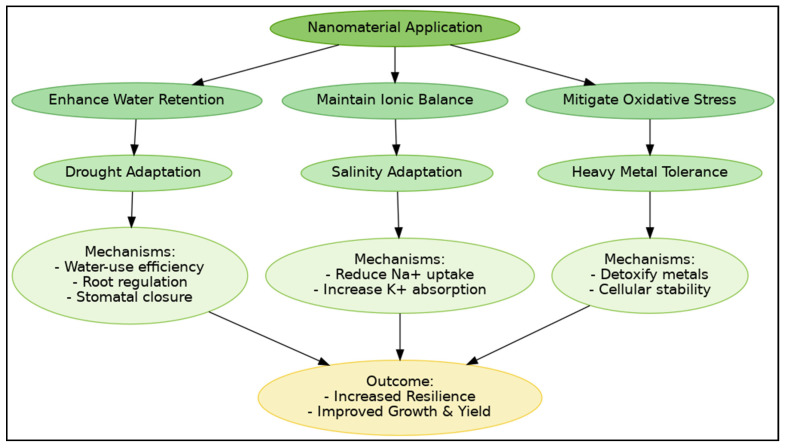
Enhancement of plant stress tolerance through nanomaterial application.

Abiotic stress caused by environmental factors, such as drought, salinity, heavy metal toxicity, and extreme temperatures, presents significant challenges to plant productivity and survival [114]. Nanomaterials have demonstrated an extraordinary ability to help plants adapt to abiotic challenges such as drought, salinity, and heavy metal toxicity. Nanomaterials, including ZnO NPs, CeO_2_ NPs, and CNTs, modulate physiological and molecular pathways to improve water retention, maintain ionic balance, and reduce oxidative stress [115]. For instance, in drought-stressed plants, nanomaterials enhance water use efficiency through mechanisms such as the regulation of root water channels and stomatal closure, processes often mediated by hormonal changes [116]. Similarly, nanomaterials help maintain ion homeostasis under salinity stress by reducing sodium uptake and increasing potassium absorption, thereby preserving cellular stability. These effects are often mediated through specific changes in gene expression and the modulation of hormones like ABA, which play crucial roles in stress adaptation. Figure 3 illustrates how these processes contribute to plant stress resistance [90].

### 5.1. Mitigating Drought and Salinity Stress

#### 5.1.1. Mechanisms of Drought Tolerance

Drought stress limits plant growth by reducing water availability and triggering oxidative stress, leading to cellular damage [117]. Nanomaterials mitigate drought stress through mechanisms that improve water use efficiency and enhance root water absorption [118]. For instance, carbon-based nanomaterials such as CNTs create nanochannels in root cell membranes, facilitating the rapid uptake and transport of water [38]. Polymeric nanomaterials such as hydrogels loaded with NPs act as water reservoirs, gradually releasing water to plants under drought conditions [116]. Additionally, NPs such as ZnO and TiO_2_ reduce oxidative damage by scavenging reactive oxygen species (ROS), thereby preventing cellular degradation during periods of water scarcity [119]. Recent studies show that nanomaterials influence the expression of drought-responsive genes such as DREB2 and AREB1, which enhance the ability of plants to cope with water stress [120].

#### 5.1.2. Mechanisms of Salinity Tolerance

Salinity stress disrupts plant growth by inducing ion toxicity, osmotic stress, and nutrient uptake imbalances [121]. Nanomaterials enhance salinity tolerance by maintaining ionic balance and regulating osmotic pressure [122]. Ag NPs, for example, mitigate ion toxicity by reducing sodium uptake while increasing potassium assimilation, thus preserving ionic homeostasis within cells [123]. Silicon (Si) NPs strengthen cell walls and improve membrane stability, thereby mitigating salt-induced dehydration [124]. Furthermore, carbon-based nanomaterials promote the production of osmolytes such as proline, which helps maintain cellular turgor and enzymatic activity, enhancing plant resilience under saline conditions [125].

### 5.2. Tolerance to Heavy Metal Stress

#### 5.2.1. Nanoparticle-Assisted Remediation

Heavy metal contamination of soil, including cadmium, lead, and arsenic, poses significant challenges to plant growth and food safety [126]. NPs help mitigate heavy metal toxicity by binding to metal ions and reducing their bioavailability [127]. Fe_2_O_3_ NPs, for instance, exhibit a high affinity for heavy metal ions, immobilizing them in the soil and preventing plant uptake [128]. Similarly, magnesium oxide NPs neutralize toxic metal ions by adsorption, rendering the soil environment less hostile to plant growth. By detoxifying the soil, these NPs improve the overall quality of the growing medium [129].

#### 5.2.2. Detoxification of Heavy Metals

Once absorbed, heavy metals cause oxidative stress, disrupt enzymatic activity, and damage cellular structures [130]. Nanomaterials mitigate these effects by enhancing plant antioxidant defense systems and promoting metal detoxification [10]. ZnO and cerium oxide (CeO_2_) NPs reduce ROS accumulation caused by heavy metal toxicity, thereby minimizing oxidative damage to cellular components [131]. Furthermore, certain NPs stimulate the synthesis of chelating agents, such as phytochelatins, which bind to heavy metal ions and sequester them in vacuoles, effectively detoxifying the cytosol. Nanomaterials also regulate gene expression related to metal uptake, including genes encoding metal transporters such as MTP1 and IRT1, which are involved in metal sequestration and detoxification [132]. This mechanism not only reduces the toxic impact of heavy metals but also helps plants maintain their metabolic balance under contaminated conditions [133]. Research has also highlighted the potential of nanopesticides, such as nanoatrazine, in enhancing plant tolerance to heavy metal stress by inhibiting photosystem II activity in plants, leading to improved stress resistance and detoxification of harmful substances [134].

### 5.3. Adaptation to Extreme Temperature Stress

#### 5.3.1. Heat Stress Resistance

Heat stress negatively affects plant metabolism, photosynthesis, and cellular stability, often leading to reduced productivity [135]. Nanomaterials enhance heat stress tolerance by stabilizing membranes and boosting antioxidant activity [10]. For example, Si NPs stimulate the production of heat shock proteins (HSPs), which protect cellular proteins and membranes from denaturation at high temperatures [136]. Metallic NPs, such as Au and Ag, help reduce heat-induced ROS accumulation and shield plants from oxidative damage [137]. Additionally, polymeric nanomaterials reflect excess solar radiation, providing a cooling effect that reduces the impact of heat stress on leaves and other exposed tissues [138].

#### 5.3.2. Cold Stress Resistance

Cold stress disrupts enzymatic activity and damages cellular membranes by inducing ice crystal formation [139]. Nanomaterials improve cold stress tolerance by stabilizing cell structures and enhancing antifreeze mechanisms [140]. For instance, graphene oxide NPs strengthen cell walls and increase membrane fluidity, preventing physical damage caused by freezing temperatures [140]. Si NPs promote the accumulation of osmolytes, such as sugars and amino acids, which lower the freezing point of intracellular fluids and reduce ice formation [141]. Furthermore, NPs enhance the activity of cold-responsive enzymes, ensuring their metabolic stability under low-temperature conditions [142]. By leveraging these mechanisms, nanomaterials can equip plants with improved tolerance to a wide range of abiotic stresses, enhancing their survival and productivity under challenging environmental conditions [39].

## 6. Mechanisms Underpinning Stress Tolerance at the Molecular and Physiological Levels

Nanomaterials contribute to plant stress tolerance by intricately interacting with molecular and physiological systems that support survival under adverse conditions. These interactions include modulating oxidative stress responses, altering gene expression, and regulating hormone balance to create a robust framework for stress resilience [15]. Nanomaterials strengthen plants’ ability to cope with environmental challenges such as drought, salinity, heavy metal contamination, and extreme temperatures by regulating ROS levels, activating signaling pathways, and promoting osmolyte accumulation [143]. Although the benefits of nanomaterials are evident, their long-term ecological impact remains a critical concern. Research into the persistence of nanomaterials in soils, water systems, and their potential bioaccumulation in plants and animals is needed to fully evaluate their environmental footprint [144]. Understanding these mechanisms would provide a foundation for optimizing the application of nanotechnology in agriculture while mitigating environmental risks and phytotoxicity. Table 3 presents a detailed summary of these mechanisms, along with the nanomaterials, their observed effects, and the associated benefits. This table highlights the multifaceted role of nanomaterials in enhancing plant resilience and recovery from abiotic stresses.

### 6.1. ROS Regulation and Antioxidant Activity

#### 6.1.1. ROS Modulation

**Figure 4:**
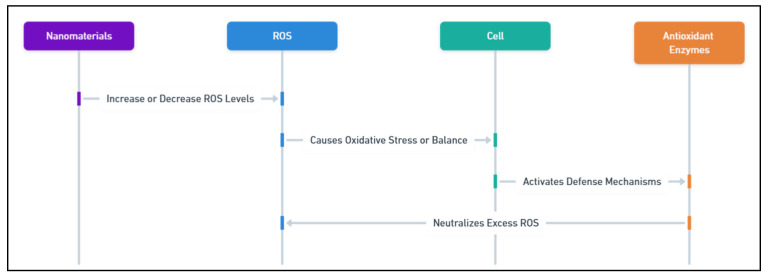
The role of nanomaterials in modulating ROS levels and enhancing antioxidant defense in plant cells.

Abiotic stress conditions, such as drought, salinity, and extreme temperatures, lead to ROS overproduction in plants, causing oxidative stress that damages cellular components, including proteins, lipids, and DNA [148]. Nanomaterials effectively mitigate oxidative stress by modulating ROS levels and stabilizing the cellular redox balance [149]. As shown in Figure 4, nanomaterials can either increase or decrease ROS levels, influencing oxidative stress and cell function. CeO_2_ NPs are particularly notable for mimicking the activities of CAT and SOD enzymes. By alternating between Ce^3+^ and Ce^4+^ oxidation states, these NPs neutralize superoxide radicals and hydrogen peroxide (H_2_O_2_), protecting cellular structures from oxidative damage [150]. Similarly, TiO_2_ NPs reduce ROS formation by stabilizing electron transport chains in chloroplasts and mitochondria, major sites of ROS generation during stress. This modulation prevents cellular damage while maintaining metabolic stability and energy production [151].

#### 6.1.2. Enhancement of Antioxidant Defenses

Nanomaterials enhance plants’ intrinsic antioxidant defense systems, enabling more effective responses to oxidative stress. Metallic NPs, such as ZnO and Ag, upregulate the activity of enzymatic antioxidants, including SOD, CAT, and peroxidase (POD) [152]. These enzymes are essential for detoxifying ROS and maintaining cellular homeostasis under stress conditions. For instance, ZnO NPs enhance SOD activity, which converts superoxide radicals into H_2_O_2_, a less reactive ROS, which is subsequently broken down by CAT into water and oxygen [153]. In addition, carbon-based nanomaterials like graphene oxide stimulate the synthesis of nonenzymatic antioxidants such as glutathione and ascorbate. These molecules directly neutralize ROS and regenerate oxidized forms of enzymatic antioxidants, ensuring sustained antioxidant capacity in stressed plants [154].

### 6.2. Gene Expression and Signaling Pathways

#### 6.2.1. Stress-Related Gene Expression

Nanomaterials significantly contribute to plant stress tolerance by modulating gene expression. Through interactions with signaling networks, nanomaterials activate stress-related genes that enhance resilience [155]. For example, ZnO NPs upregulate genes encoding aquaporin, improving water transport across cell membranes and increasing water use efficiency under drought conditions [156]. Similarly, Si NPs influence the expression of lignin biosynthesis genes, strengthening cell walls and providing structural defenses against both abiotic and biotic stresses. These genetic responses allow plants to adapt their physiological and metabolic processes to environmental challenges, improving survival and productivity under stress [157].

#### 6.2.2. Activation of Stress Response Pathways

Nanomaterials are pivotal in activating stress response pathways involving key signaling molecules and cascades. Metallic NPs, such as Fe_2_O_3_, enhance calcium signaling, a critical component of stress perception in plants [10]. Increased cytosolic calcium levels induced by these NPs activate downstream kinases and transcription factors that regulate stress-responsive genes [158]. Similarly, carbon-based nanomaterials such as graphene oxide facilitate the production of secondary messengers like nitric oxide (NO) and H_2_O_2_, which modulate oxidative stress responses and osmotic balance pathways. These activated signaling pathways enhance the ability of plants to perceive, respond to, and recover from stress, promoting improved adaptation and resilience [159].

### 6.3. Hormonal Balance and Osmolyte Accumulation

#### 6.3.1. Influence on Plant Hormones

Nanomaterials regulate the synthesis, transport, and signaling of plant hormones that are vital for stress adaptation [116]. Hormones such as ABA, auxins, gibberellins, cytokinins, and ethylene play distinct roles in modulating plant responses to abiotic stress [160]. For example, Ag NPs increase ABA levels, inducing stomatal closure to conserve water during drought stress [161]. CNTs enhance auxin transport, promote root elongation, and improve water and nutrient uptake under water-limited conditions [162]. Nanomaterials also balance gibberellin and cytokinin levels to prioritize growth while complementing stress responses [163]. For instance, NPs can elevate cytokinin levels in shoots to maintain photosynthetic activity while simultaneously increasing auxin concentrations in roots to enhance water absorption [164]. To ensure the responsible application of these materials in agricultural settings, regulatory guidelines must be established to govern their safe use and prevent potential hormone imbalance or unintended consequences in crops and ecosystems.

#### 6.3.2. Osmoprotectants and Stress Tolerance

The accumulation of osmoprotectants, such as proline, glycine betaine, and soluble sugars, is a key mechanism for maintaining osmotic balance and protecting cellular structures under stress conditions [165]. Nanomaterials stimulate the synthesis and accumulation of osmolytes, which stabilize proteins, membranes, and other cellular components [166]. For instance, Si NPs enhance proline biosynthesis by acting both as an osmolyte and an ROS scavenger, mitigating oxidative damage [141]. Similarly, carbon-based nanomaterials promote the accumulation of trehalose, a sugar that protects cellular machinery against dehydration and freezing damage. These osmolytes help plants retain water, stabilize their enzymatic activity, and sustain metabolic processes during abiotic stress [38]. Despite the promising results, the environmental fate of nanomaterials and their long-term effects on soil health and microbial ecosystems need further investigation to ensure their sustainable use [167].

By influencing ROS regulation, gene expression, hormonal balance, and osmolyte accumulation, nanomaterials provide plants with a multifaceted defense mechanism against environmental stresses. These interactions underscore the vast potential of nanotechnology to enhance agricultural productivity despite climate change and resource limitations [15,168].

## 7. Environmental and Health Implications of Nanomaterials in Agriculture

Nanomaterials hold great promise for improving agricultural practices, but their widespread use also presents challenges that must be addressed. As with any emerging technology, the long-term effects on both the environment and human health must be studied thoroughly. Key concerns include the persistence of nanomaterials in the soil, potential bioaccumulation, and their effects on non-target organisms, including soil microorganisms, insects, and aquatic life [35]. To minimize these risks, it is essential to develop biodegradable nanomaterials that degrade into non-toxic byproducts. Additionally, implementing a lifecycle approach to assess the environmental impact of nanomaterials is critical [169]. Regulatory frameworks must be updated to address the unique risks posed by nanomaterials, including standardized testing for environmental safety and risk assessments. The use of green synthesis methods that produce biodegradable nanomaterials, alongside the development of precise application systems like nanosensors, can help mitigate these risks. These advancements should be accompanied by public awareness initiatives to ensure the safe and responsible application of nanomaterials in agriculture [35,170].

**Figure 5:**
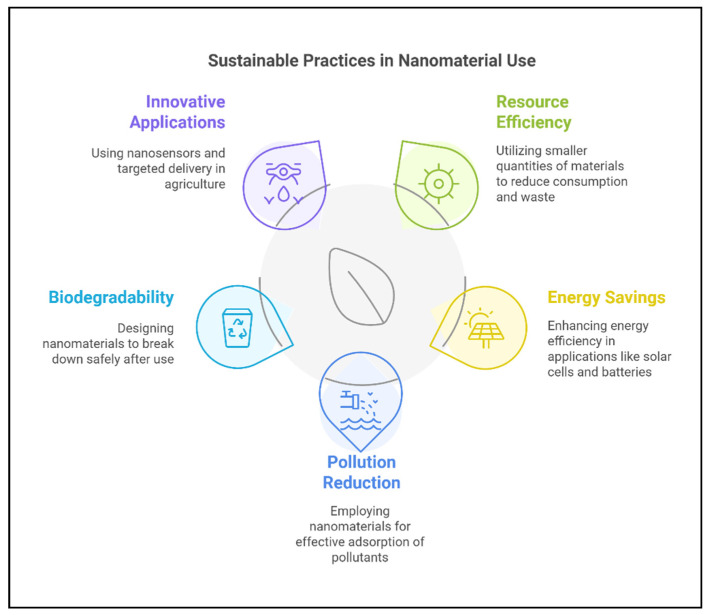
Sustainable practices in nanomaterial application for agriculture.

**Figure 6:**
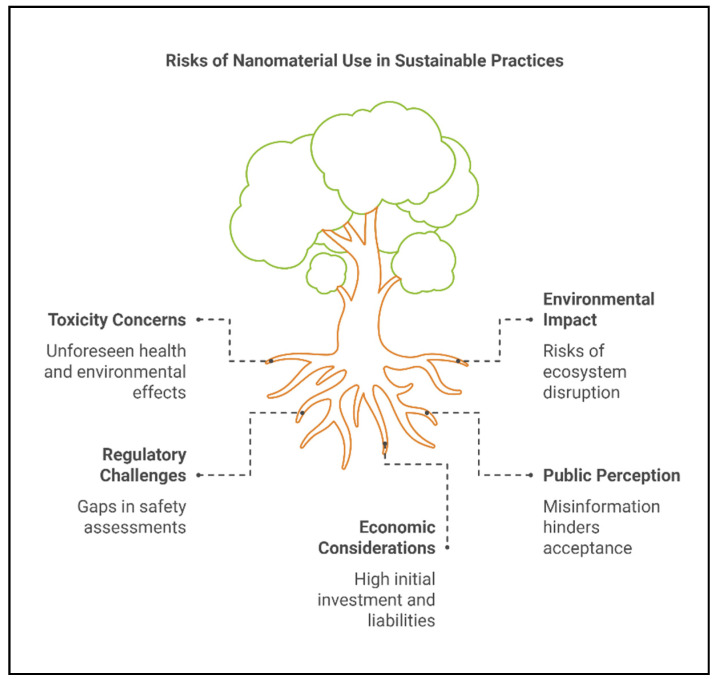
Potential environmental and health risks of nanomaterial use in agriculture.

The application of nanomaterials in agriculture has immense potential to revolutionize farming practices, offering sustainable solutions to enhance crop growth and mitigate stress. However, their widespread use raises critical environmental and health concerns [171]. Unintended consequences, such as soil and water contamination, ecosystem imbalances, and toxicity to non-target organisms, must be carefully addressed [172]. A comprehensive understanding of the environmental behavior, risk mitigation strategies, and regulatory frameworks of nanomaterials is essential to ensure their safe and sustainable use. Figure 5 summarizes sustainable practices for nanomaterial applications, and Figure 6 highlights potential risks associated with their use.

### 7.1. Ecotoxicological Concerns and Potential Risks

#### 7.1.1. Accumulation in Soil and Water

The accumulation of nanomaterials in agricultural soils and water systems represents a significant environmental concern. After application, nanomaterials can persist in soil matrices and alter soil structure, nutrient availability, and microbial communities [173]. For instance, metallic NPs such as Ag and ZnO, which are beneficial to plant growth, may accumulate over time and affect the physical and chemical properties of soil. These NPs can disrupt microbial ecosystems, negatively impacting the microbes that are essential for nutrient cycling and organic matter decomposition [22]. Additionally, nanomaterials may influence plant-associated microorganisms in the rhizosphere, with some nanoparticles stimulating beneficial microbial activity, enhancing nutrient availability and soil health [174]. Furthermore, runoff containing nanomaterials can contaminate water bodies, leading to nanoparticle bioaccumulation in aquatic ecosystems. This not only threatens aquatic life but also poses risks to human health through the food chain [175].

#### 7.1.2. Potential Plant and Ecosystem Toxicity

Nanomaterials, depending on their composition and concentration, can exhibit phytotoxic effects on plants and toxicity to non-target organisms within the ecosystem. Excessive concentrations of metallic NPs such as Ag and Cu may induce oxidative stress in plants, causing cellular damage and reduced growth [176]. In addition to their direct effects on plants, nanomaterials can enter the food web through soil fauna and plant tissues, impacting higher trophic levels [177]. For instance, carbon-based NPs inhibit seed germination and root elongation in sensitive plant species, emphasizing the need for species-specific toxicity assessments. These unintended consequences highlight the importance of dose optimization and the thorough evaluation of nanomaterial formulations to minimize ecological risks [178].

### 7.2. Sustainable Use and Biodegradability of Nanomaterials

#### 7.2.1. Development of Eco-Friendly Nanomaterials

To address concerns regarding environmental accumulation and toxicity, the development of eco-friendly and biodegradable nanomaterials has become a priority. Biodegradable nanomaterials, such as those derived from chitosan, cellulose, or other biopolymers, offer environmentally benign alternatives to conventional NPs. These materials degrade naturally into harmless byproducts, reducing their environmental persistence [179]. For instance, chitosan-based NPs enhance plant growth and stress tolerance while minimizing ecological risks because of their biocompatibility and biodegradability [180]. Recent studies have also demonstrated that chitosan-based nanoparticles, combined with other growth-promoting compounds, can enhance soybean germination and seedling growth under stress conditions [181]. Furthermore, green synthesis methods that use plant extracts, microorganisms, or other biological agents provide a sustainable approach to nanoparticle production, eliminating the need for hazardous chemicals [182].

#### 7.2.2. Strategies for Reducing Environmental Impact

Implementing strategies to reduce the environmental impact of nanomaterials is essential for their sustainable application in agriculture. The encapsulation of NPs within polymeric or lipid-based carriers enhances their stability and effectiveness while minimizing environmental release [183]. Controlled-release formulations enable the precise delivery of nutrients or pesticides, reducing waste and contamination [184]. Additionally, integrating nanomaterials with precision agriculture technologies, such as nanosensors, ensures targeted application based on real-time soil and plant health monitoring. These strategies, coupled with effective waste management practices, significantly mitigate the environmental risks associated with nanomaterials [185].

### 7.3. Regulatory and Safety Guidelines

#### 7.3.1. Current Regulations on Nanomaterial Use

Despite the rapid advancements in nanotechnology, the regulatory frameworks governing the use of nanomaterials in agriculture remain underdeveloped in many regions [35]. Existing regulations often focus on general chemical safety without fully addressing the unique properties and risks associated with NPs. For instance, although the European Union’s REACH (Registration, Evaluation, Authorization, and Restriction of Chemicals) framework includes provisions for nanomaterials, these guidelines are still evolving [186]. Similarly, in countries like the United States, agencies such as the Environmental Protection Agency (EPA) and the Food and Drug Administration (FDA) are beginning to evaluate the environmental and health implications of nanomaterials. However, the absence of standardized testing protocols and comprehensive long-term safety data poses significant challenges to effective regulatory oversight [187].

#### 7.3.2. Recommendations for Safe Application

To ensure the safe use of nanomaterials in agriculture, comprehensive safety assessments and standardized guidelines are urgently needed. Key recommendations include conducting detailed ecotoxicological studies to evaluate the long-term impacts of nanomaterials on soil, water, and nontarget organisms [177]. Establishing permissible concentration limits and implementing labeling requirements for nanomaterial-based agricultural products are critical steps to mitigate potential risks. Collaborative efforts between researchers, industry stakeholders, and regulatory agencies are essential for developing best practices for the production, application, and disposal of nanomaterials [188]. Additionally, public awareness campaigns can play a pivotal role in promoting the responsible use of nanotechnology in agriculture, ensuring its benefits are realized without compromising environmental and human health [189].

By addressing environmental and health concerns through sustainable practices and robust regulations, the transformative potential of nanomaterials in agriculture can be responsibly harnessed, ensuring increased productivity while preserving ecological integrity [170].

## 8. Future Prospects and Research Directions

The application of nanotechnology in agriculture is poised for transformative breakthroughs, with the potential to revolutionize traditional farming practices while addressing critical challenges related to food security, climate change, and environmental sustainability [190]. Future advancements in nanomaterials will require improvements in synthesis methods, integration with emerging technologies such as the IoT and AI and filling knowledge gaps to ensure safe and effective use. Continued research and innovation in these areas will shape the future trajectory of nanotechnology in agriculture, fostering a balance between productivity and ecological health [191]. Key challenges for future research include the need for long-term ecological impact studies, improvements in the scalability of nanomaterial production, and better regulatory frameworks for safe applications. These challenges should be addressed through interdisciplinary collaborations and increased focus on understanding the persistence and bioaccumulation of nanomaterials in environmental systems. A summary of emerging trends, innovations, benefits, and challenges is presented in Table 4.

### 8.1. Advances in Nanomaterial Synthesis and Functionalization

#### 8.1.1. Innovations in Synthesis

The synthesis of nanomaterials has advanced significantly, but further innovations are needed to meet the specific demands of agriculture [196]. Green synthesis methods using biological systems such as plant extracts, fungi, and bacteria are gaining popularity as eco-friendly alternatives to conventional chemical synthesis. These methods reduce the use of toxic reagents and lower the environmental footprint of nanomaterial production [197]. For instance, Ag NPs synthesized from plant extracts exhibit strong antimicrobial properties, making them ideal for pest and disease management. Additionally, advances in green synthesis can enable cost-effective production, facilitating wider adoption in resource-limited settings [198].

In addition to green synthesis, scalable methods for producing NPs with uniform size, shape, and composition are essential. Techniques such as microfluidics and nanolithography can precisely control the characteristics of NPs, enhancing their performance in agricultural applications [199]. Moreover, hybrid nanomaterials that combine multiple functionalities—such as nutrient delivery, pathogen control, and stress mitigation—are emerging as versatile tools for addressing complex agricultural challenges [20]. However, the scalability of these synthesis methods remains a challenge, as does reducing the cost of production to make nanomaterials more accessible to farmers globally. Overcoming these barriers will be key to the widespread adoption of nanotechnology in agriculture.

#### 8.1.2. Targeted Functionalization for Agriculture

Functionalizing nanomaterials by modifying their surface properties is critical for enhancing their specificity, stability, and efficiency. In agriculture, targeted functionalization ensures that nanomaterials interact effectively with plant systems while minimizing unintended effects [200]. For example, coating NPs with biopolymers like chitosan improves biocompatibility and enables the controlled release of nutrients or agrochemicals. Similarly, functionalizing NPs with ligands or biomolecules facilitates targeted delivery to specific plant tissues, such as roots or leaves, thereby optimizing their effectiveness [201].

Recent advancements in functionalization include the development of smart nanomaterials that respond to environmental cues such as pH, temperature, and moisture changes. These responsive materials release their payloads only under specific conditions, thereby reducing wastage and environmental contamination [13]. Such innovations not only enhance the efficiency of nanomaterials but also align with the principles of precision agriculture, positioning them as integral components of sustainable farming’s future.

### 8.2. Integration with Precision Agriculture and Smart Technologies

#### 8.2.1. IoT- and AI-Enabled Applications

The integration of nanotechnology with IoT and AI offers transformative opportunities for precision agriculture, enabling real-time monitoring, data-driven decision-making, and resource optimization [202]. Nanosensors capable of detecting soil moisture, nutrient levels, or pathogen presence at the nanoscale can be connected to IoT networks to provide farmers with actionable insights. When combined with AI algorithms, these sensors can analyze large datasets to predict crop health trends, recommend timely interventions, and optimize resource use [13]. For example, nanosensors embedded in soil can continuously monitor nutrient availability and trigger the release of nanomaterial-based fertilizers when needed, ensuring efficient nutrient management [203].

AI-powered platforms also enhance the design and deployment of nanomaterials by simulating their interactions with plant systems and predicting their long-term effects. This approach reduces reliance on trial-and-error methods, thereby accelerating the development of effective solutions [204]. Furthermore, IoT-enabled drones equipped with nanosensors can map large agricultural fields, identify stress hotspots, and deliver nanomaterials with precision, minimizing wastage and reducing environmental impact [205].

The application of nanotechnology in precision agriculture can enhance traditional farming practices. By integrating nanomaterials with emerging technologies like drones, sensors, and AI, farmers can achieve more efficient resource management and better crop management [202]. Nanosensors connected to IoT devices continuously monitor soil conditions, nutrient levels, and plant health, enabling precise interventions. For example, nanosensors can detect nutrient deficiencies and trigger the release of nanomaterial-based fertilizers only when required, reducing wastage and ensuring optimal nutrient uptake by plants [13,206]. Additionally, nanosensors can detect environmental stressors and alert farmers about emerging issues, allowing for early interventions to minimize damage and improve productivity [207].

Drones equipped with nanomaterial delivery systems can efficiently apply fertilizers, pesticides, and growth regulators precisely where needed. This targeted delivery ensures minimal environmental impact and maximizes resource efficiency [208]. Moreover, drones can monitor large agricultural areas, providing real-time data on crop health, moisture levels, and pest infestations. This capability enables farmers to take proactive actions and avoid overuse of resources, such as water and chemicals, while enhancing sustainability [209].

#### 8.2.2. Precision Delivery of Nanomaterials

Precision delivery systems are integral to sustainable nanotechnology in agriculture, ensuring that nanomaterials reach their target sites efficiently with minimal loss [202]. Advances in encapsulation technologies, such as nanoemulsions and liposomes, have facilitated the development of controlled-release formulations that deliver nutrients or pesticides over extended periods. These systems reduce the frequency of applications, lowering labor and material costs while minimizing environmental contamination [210].

In addition to controlled release, the use of environmentally responsive nanocarriers ensures that nanomaterials are released only under specific conditions, such as changes in temperature or pH [211]. For instance, pH-sensitive nanocarriers release fertilizers in response to soil acidity, improving nutrient availability while reducing leaching into water bodies. Such precision delivery technologies not only enhance the efficiency of nanomaterials but also align with global efforts to promote sustainable and resource-efficient agricultural practices [212].

### 8.3. Addressing Research Gaps and Long-Term Effects

#### 8.3.1. Long-Term Ecological Impact Studies

Despite the promising potential of nanotechnology in agriculture, significant research gaps remain, particularly concerning the long-term ecological impacts of nanomaterials. Current studies have primarily focused on short-term effects, leaving critical questions about the persistence, bioaccumulation, and toxicity of nanomaterials unanswered [213]. For example, the fate of NPs in soil ecosystems and their interactions with microbial communities require detailed investigation. Understanding these dynamics is essential for developing strategies to mitigate potential risks, such as soil degradation or the disruption of beneficial microbial processes [214]. Future research should emphasize the long-term impact of nanomaterials on ecosystems, including their potential effects on non-target organisms and the environment. Long-term field studies are also necessary to evaluate the cumulative effects of repeated nanomaterial applications on plant health, soil quality, and ecosystem balance. These studies should consider not only the direct impacts on target crops but also the indirect effects on non-target organisms, including pollinators, soil fauna, and aquatic ecosystems [215]. Addressing these gaps will enable researchers to build a comprehensive risk assessment framework to guide the safe and sustainable use of nanomaterials in agriculture.

#### 8.3.2. Future Research Directions

Future research in agricultural nanotechnology should prioritize the development of multifunctional nanomaterials capable of addressing multiple challenges simultaneously [200]. For example, hybrid NPs designed to deliver nutrients while protecting against pathogens and enhancing stress tolerance could streamline agricultural inputs, reducing costs and environmental impact. Additionally, the application of nanomaterials in emerging agricultural systems, such as vertical farming, hydroponics, and aquaponics, presents unique opportunities for precision and efficiency, particularly in resource-limited environments [216].

Collaborative efforts between academia, industry, and policymakers are essential for translating research findings into practical applications. Investments in interdisciplinary research that integrates nanotechnology with plant science and environmental studies can drive innovation and ensure that nanotechnology contributes to sustainable and resilient agricultural systems. Furthermore, public engagement and education will play pivotal roles in fostering the acceptance of nanotechnology, addressing concerns, and highlighting its potential to revolutionize agriculture [217]. The focus should be on ensuring scalability, cost-effectiveness, and minimizing any unintended long-term ecological impacts through a sustainable approach to research and application. By advancing synthesis methods, integrating nanotechnology with smart technologies, and addressing critical research gaps, the future of agricultural nanotechnology holds immense promise. These efforts will pave the way for innovative solutions that enhance productivity, minimize environmental impact, and support global food security.

## 9. Conclusions and Final Remarks

Nanotechnology has emerged as a transformative force in agriculture, offering innovative solutions to enhance plant growth, improve stress tolerance, and address the pressing challenges of modern farming. Through the development and application of nanomaterials, agriculture has entered a new era characterized by precision, efficiency, and sustainability. These advanced materials, with unique properties such as high surface area, reactivity, and the ability to interact at the molecular level, have shown immense potential for improving various aspects of plant development. From enhancing seed germination and root growth to boosting photosynthetic efficiency and nutrient metabolism, nanomaterials provide versatile tools for optimizing crop productivity. Additionally, their role in mitigating abiotic stresses—such as drought, salinity, and extreme temperatures—underscores their critical importance in ensuring agricultural resilience amid climate change and environmental challenges.

At the molecular level, nanomaterials regulate ROS, enhance antioxidant defenses, and modulate stress-related gene expression and signaling pathways. These mechanisms allow plants to adapt to adverse conditions by maintaining cellular integrity, stabilizing metabolic processes, and activating robust defense responses. Moreover, nanomaterials influence hormonal balance and promote the accumulation of osmoprotectants, which are vital for water retention, membrane stabilization, and osmotic balance under stress. The precision with which they deliver nutrients and agrochemicals, combined with advancements in functionalization and controlled-release systems, further highlights their efficiency and sustainability. By integrating nanotechnology with cutting-edge smart technologies such as the IoT and AI, precision agriculture has been elevated to unprecedented levels. This integration facilitates real-time monitoring, resource optimization, and targeted interventions, providing farmers with powerful tools to improve productivity and sustainability.

Despite the remarkable advancements in nanotechnology, its widespread application in agriculture requires careful consideration of its environmental and health implications. The accumulation of nanomaterials in soil and water ecosystems poses risks to non-target organisms and microbial communities, potentially disrupting ecological balance. Addressing these concerns necessitates the development of eco-friendly and biodegradable nanomaterials and the implementation of controlled application strategies to minimize environmental impacts. Regulatory frameworks and safety guidelines must evolve to address the unique challenges posed by nanotechnology, ensuring responsible use while fostering innovation. Collaborative efforts among researchers, policymakers, and industry stakeholders are essential for the safe and effective integration of nanotechnology into agricultural systems.

The future of nanotechnology in agriculture holds immense promise, but its success depends on addressing critical research gaps and advancing understanding of its long-term effects. Multifunctional nanomaterials tailored for specific agricultural applications present exciting opportunities to streamline inputs, improve efficiency, and reduce costs. Long-term field studies are crucial for evaluating the ecological and health implications of repeated nanomaterial use, ensuring that their benefits are not overshadowed by unintended consequences. Furthermore, public engagement and education will play a vital role in fostering the acceptance of nanotechnology, dispelling misconceptions and promoting its potential to address global food security challenges.

In conclusion, nanotechnology can redefine the agricultural landscape by delivering sustainable and scalable solutions to agricultural challenges. By harnessing agricultural transformative capabilities, we can move toward a future characterized by increased productivity, reduced environmental impact, and enhanced resilience to climate change. Through continued innovation, interdisciplinary collaboration, and a commitment to sustainability, nanotechnology will play a pivotal role in shaping a secure and sustainable agricultural system for future generations.

## Figures and Tables

**Table 1:** Classification and characteristics of nanomaterials used in plant sciences.

Category	Examples	Key Applications	Key Properties	References
Metallic NPs	Ag, Au, ZnO, and Fe_2_O_3_	Antimicrobial activity, nutrient delivery, chlorophyll synthesis, pathogen control, and improved micronutrient availability	High reactivity, controlled release behavior, and efficient nutrient uptake	[30,31]
Carbon-Based Nanomaterials	CNTs, graphene oxide, and fullerenes	Enhancing water and nutrient uptake, modulating oxidative stress, efficient agrochemical delivery, and biosensing applications	Structural stability, electrical and mechanical properties, and creation of nanochannels in roots for better absorption	[32,33]
Polymeric Nanomaterials and Composites	Chitosan-based NPs, polymeric composites	Targeted and slow release of nutrients, pesticides, or growth regulators; environmentally friendly pest management; reduced ecological footprint	Biodegradable, biocompatible, and antimicrobial properties	[34,35]

**Table 2:** Effects of nanomaterials on plant growth parameters.

Nanomaterial Type	Specific Effects on Plant Growth	Key Parameters Affected	References
Graphene-Family Nanomaterials (GFNs)	Enhanced photosynthesis, stress tolerance, root elongation, and germination.	Root elongation and photosynthetic efficiency	[80,106]
TiO_2_ NPs	Improved photosynthetic rate and seedling growth; enhanced light absorption.	Photosynthetic efficiency and seedling growth	[107]
Ag NPs	Increased root elongation and pathogen resistance.	Root elongation and biomass production	[6]
ZnO NPs	Stimulated chlorophyll production and improved biomass under stress conditions.	Photosynthetic efficiency, biomass	[108]
CNTs	Enhanced water uptake and nutrient translocation; creation of nanochannels in root membranes.	Root elongation and nutrient uptake	[109]
Chitosan-Based Nanomaterials	Improved germination rates and overall plant vigor under abiotic stress.	Germination and stress tolerance	[110,111]
Fullerenes	Modulated oxidative stress and increased seedling growth.	Stress tolerance and seedling growth	[112]
Engineered NPs	Enhanced growth under abiotic stress; promoted uptake of macronutrients, such as nitrogen and phosphorus.	Biomass production and nutrient uptake	[113]
Au NPs	Increased biomass and improved antioxidant activity in plants exposed to environmental stress.	Biomass production and stress resilience	[110]

**Table 3:** Molecular and physiological mechanisms influenced by nanomaterials in stress adaptation.

Nanomaterial	Mechanism	Observed Effects	Key Benefits	References
CeO_2_ NPs	ROS Scavenging	Reduction in ROS levels and prevention of oxidative damage	Protects cells against oxidative stress under abiotic conditions	[137]
AgNPs	Antioxidant Enzyme Activation	Increased activity of catalase (CAT) and superoxide dismutase (SOD)	Strengthens the plant’s intrinsic antioxidant defense system	
CNTs	Osmolyte Accumulation	Enhanced accumulation of proline and soluble sugars	Improves osmotic balance and cellular stability	[145]
ZnO NPs	Stress-Related Gene Upregulation	Upregulation of drought- and salinity-responsive genes	Enhances stress resilience at the molecular level	[146]
Si NPs	Hormonal Modulation	Boosted abscisic acid (ABA) levels for drought tolerance	Promotes water conservation and stress adaptation	[147]

**Table 4:** Emerging trends and future directions for nanotechnology in agriculture.

Research Area	Emerging Innovations	Expected Benefits	Current Challenges	References
Smart Nanomaterials	Stimuli-responsive nanomaterials for the controlled release of agrochemicals	Improved efficiency and reduced wastage of fertilizers and pesticides	High production costs and scalability issues	[192]
Integration with Precision Agriculture	Drone-enabled nanosensors for real-time monitoring of crop health	Enhanced resource use efficiency and optimized farming practices	Integration of nanosensors with existing technologies	[193]
Biodegradable and Eco-Friendly Nanomaterials	Green synthesis using plant extracts and microbes	Reduced environmental footprint and safer applications	Standardization of synthesis methods and regulatory acceptance	[194]
Multifunctional NPs	Hybrid NPs combining nutrient delivery, stress tolerance, and pest control	Simplified agricultural inputs, cost reduction, and enhanced effectiveness	Balancing multifunctionality with ecological safety	[195]
Long-Term Impact Studies	Field trials assessing the ecological and environmental impacts of nanomaterials	Better understanding of sustainability and safety in agricultural ecosystems	Limited availability of long-term ecological data	[50]
